# Simultaneous Transcranial Magnetic Stimulation and Functional Magnetic Resonance Imaging: Aspects of Technical Implementation

**DOI:** 10.3389/fnins.2020.554714

**Published:** 2020-09-29

**Authors:** Elisabeth C. Caparelli, Tianye Zhai, Yihong Yang

**Affiliations:** Neuroimaging Research Branch, National Institute on Drug Abuse, National Institutes of Health, Baltimore, MD, United States

**Keywords:** transcranial magnetic stimulation, functional magnetic resonance imaging, echo-planar imaging, radio frequency coil, image artifact, temporal signal-to-noise ratio

## Abstract

The simultaneous transcranial magnetic stimulation (TMS) and functional magnetic resonance imaging (fMRI) offers a unique opportunity to non-invasively stimulate brain circuits while simultaneously monitoring changes in brain activity. However, to take advantage of this multimodal technique, some technical issues need to be considered/addressed. In this work, we evaluated technical issues associated with the setup and utilization of this multimodal tool, such as the use of a large single-channel radio frequency (rf) coil, and the artifacts induced by TMS when interleaved with the echo-planar imaging (EPI) sequence. We demonstrated that good image quality can be achieved with this rf coil and that the adoption of axial imaging orientation in conjunction with a safe interval of 100 ms, between the TMS pulse and imaging acquisition, is a suitable combination to eliminate potential image artifacts when using the combined TMS-fMRI technique in 3-T MRI scanners.

## Introduction

The concurrent transcranial magnetic stimulation (TMS) and functional magnetic resonance imaging (fMRI) provides a non-invasive method for real-time evaluation of neuronal activity induced by TMS. It has the potential to identify brain areas of functional relevance to acute TMS, supporting causal brain connectivity and brain – behavior inferences across the entire brain (see Table 1). Therefore, it poses a step forward toward understanding the underlying mechanism of magnetic stimulation.

**TABLE 1:** A summary listing some of the previous TMS-fMRI work.

References	MRI system	rf coil	TMS coil	Stimulus site
Bohning et al. (1999)	Picker EDGE 1.5 T	MR head coil	Dantec MagPro non-ferromagnetic TMS coil of figure-8	Left motor cortex
Bohning et al. (2000)	Picker EDGE 1.5 T	MR head coil	Dantec MagPro non-ferromagnetic TMS coil of figure-8	Left motor cortex
Bestmann et al. (2003b)	Siemens 2 T	Standard transmit–receive head coil	Magstim non-ferromagnetic figure-of-eight coil	Left motor cortex
Bestmann et al. (2004)	Siemens Trio 3 T	MRI head coil	Magstim non-ferromagnetic figure-of-eight coil	Left sensorimotor cortex
Li et al. (2004)	Picker EDGE 1.5 T	Head coil	Dantec MagPro non-ferromagnetic TMS coil of figure-8	Left prefrontal cortex
Bestmann et al. (2005)	Siemens 2.9 T Trio	Standard transmit–receive head coil	Magstim non-ferromagnetic figure-of-eight coil	Left premotor cortex
Bestmann et al. (2006)	Siemens Trio 3 T	Transmit–receive head-coil	Magstim non-ferromagnetic figure-of-eight coil	Left motor cortex
Ruff et al. (2006)	Siemens Sonata 1.5 T	Custom visual surface coil	Magstim non-ferromagnetic figure-of-eight coil	Right FEF and vertex
Sack et al. (2007)	Siemens Trio 3 T	Standard transmit–receive head-coil	Magstim non-ferromagnetic figure-of-eight coil	Right parietal
Ruff et al. (2008)	Siemens Sonata 1.5 T	Custom-built visual surface coil	Magstim non-ferromagnetic figure-of-eight coil	Right intra-parietal sulcus
Moisa et al. (2010)	Siemens Trio 3 T	One-channel RF transmit/receive head coil	MagVenture MRI-compatible figure-8 (MRi-B88)	Left motor cortex
Caparelli et al. (2010)	Varian 4 T	One-channel RF transmit/receive head coil	Magstim non-ferromagnetic figure-of-eight coil	Visual cortex
Feredoes et al. (2011)	Siemens Sonata 1.5 T	(Not stated)	Magstim non-ferromagnetic figure-of-eight coil	Right DLPFC
Caparelli et al. (2012)	Varian 4 T	One-channel RF transmit/receive head coil	Magstim non-ferromagnetic figure-of-eight coil	Visual cortex
Hanlon et al. (2013)	Siemens Trio 3 T	12-channel head coil (RAPID Biomedical)	Magstim non-ferromagnetic figure-of-eight coil	Left DLPFC
de Weijer et al. (2014)	Philips Achieva 3 T	FLEX-L receive coils	Magstim non-ferromagnetic figure-of-eight coil	M1 and dSMG
Hanlon et al. (2016)	Siemens Trio 3 T	12-channel head coil (RAPID Biomedical)	Magstim non-ferromagnetic figure-of-eight coil	Left MPFC and left DLPFC
Jung et al. (2016)	Philips 3 T	6-channel head coil	Magstim non-ferromagnetic figure-of-eight coil	Vertex and left M1
Navarro de Lara et al. (2017)	Siemens Trio 3 T	MR coil array (7-channel)	MagVenture MRI-compatible figure-8 (MRi-B91)	Left M1
Vink et al. (2018)	Philips 3 T	MR receiver coil [FLEX-L (de Weijer et al., 2014)]	Magstim non-ferromagnetic figure-of-eight coil	Left DLPFC and M1
Dowdle et al. (2018)	Siemens Trio 3 T	12-channel head coil (RAPID Biomedical)	Magstim non-ferromagnetic figure-of-eight coil	Left DLPFC

**DLPFC, dorsolateral prefrontal cortex; FEF, frontal eyes field; MPFC, medial prefrontal cortex; M1, primary motor cortex; dSMG, dorsal part of the supramarginal gyrus.**

However, before taking advantage of this multimodal technique, some technical difficulties (Bohning et al., 1998; Bestmann et al., 2003a; Weiskopf et al., 2009; Bungert et al., 2012; Navarro de Lara et al., 2017) need to be addressed. A full assessment on passive (presence of a TMS coil) and active (during magnetic stimulation) image artifacts induced by TMS have been previously reported (Bestmann et al., 2003a), in which one of the first MRI compatible TMS coils, developed by Magstim, was used, and images were acquired on a 2-T scanner. Although new MRI-compatible TMS coils have been developed, 3-T scanners have become the primary imaging research tool, and imaging software and hardware have advanced significantly in recent years; only brief assessments have been reported lately on either passive (Bungert et al., 2012; Navarro de Lara et al., 2017) or active (Navarro de Lara et al., 2017) TMS-induced image artifacts. Therefore, a comprehensive evaluation on the use of this multimodal tool in its current state is needed.

In this work, we aim to provide an update on the technical aspects of this multimodal tool based on the latest developments of the MRI and TMS techniques. Due to the lack of inner space from most multichannel radio frequency (rf) coils, whole brain imaging acquisition may only be achieved using single-channel birdcage rf coils when combined with TMS; therefore, imaging quality associated with the use of a birdcage rf coil was accessed. Potential image artifacts (passive and active) induced by the latest version of an MRI-compatible TMS coil, on images acquired with echo-planar imaging (EPI) sequences, at a 3-T Prisma Siemens scanner, were also evaluated. Our work demonstrated that this multimodal technique can be easily used when these technical issues are addressed.

## Methods

### Phantoms and Human Participant

Two phantoms were used in the study to assess quality of images acquired from two rf coils, as well as passive and active image artifacts in the TMS-MRI setup:

1. Bottle phantom: a cylindrical plastic bottle phantom (diameter = 4.3 in, length = 7.9 in) provided by Siemens for standard costumer quality assurance (3.75 g NiSO_4_ × 6H_2_O, 5 g NaCl per 1,000 g H_2_O dist., Siemens Medical Solutions United States, Inc., Malvern, PA);
2. ACR phantom: an American College of Radiology (ACR) MRI phantom (diameter = 8 in, length = 6.82 in., J. M. Specialty Parts Inc. San Diego, CA).

A healthy adult (male, 25 years of age) participated in this study. The participant gave written informed consent approved by the institutional review board of the National Institute on Drug Abuse.

### Data Acquisition

#### MRI Scanning

Images were acquired at a 3-T Prisma Siemens system. A transmit-receive (Tx/Rx) single-channel birdcage head rf coil and a 20-channel head rf coil were used for image quality evaluation (rf coil comparison). Images acquired with the 20-channel coil had either parallel imaging (IPAT) ON (acceleration factor = 2) or OFF, whereas parallel image was not available for the Tx/Rx single-channel coil. Images acquired with the 20-channel coil had prescan normalize ON, but those acquired with the Tx/Rx-coil had it OFF. FMRI data were acquired using a single-shot gradient-echo (GRE) echo-planar imaging (EPI) sequence.

#### rf Coil Comparison

EPI scans were performed on the bottle phantom and the ACR phantom with the following imaging parameters:

1. Bottle phantom: TE/TR(20-ch)/TR(Tx/Rx) = 27/2,000/2,130 ms, in-plane resolution 3.4 × 3.4 × 4 mm^3^, 39 slices (Tx/Rx and 20-channel – IPAT ON)/34 slices (20-channel – IPAT OFF), 100 volumes, axial orientation;
2. ACR phantom: TE(20ch)/TE(Tx/Rx)/TR = 27/20/2,000 ms, in-plane resolution 3.4 × 3.4 × 4 mm^3^, 39 slices (20-channel – IPAT ON), 20 volumes, axial orientation.

#### TMS-Induced Image Artifacts

Further data acquisition to evaluate the passive and active image artifacts induced by TMS were conducted with the Tx/Rx head coil, since it is the only commercially available volume coil that can fit the TMS coil and its holder inside, along with the scanning object: either the bottle phantom or the participant’s head. The following imaging parameters were used: echo time (TE)/repetition time (TR) of 27/2,500 ms, tr-delay of 500 ms, in-plane resolution of 3.4 × 3.4 × 4.4 mm^3^, 36 slices per volume, and 20 volumes were acquired to evaluate passive artifacts (with the phantom and the participant) and 50 volumes were acquired to evaluate active image artifacts with the bottle phantom. The anatomical image of the participant head was acquired with a high-resolution (1 × 1 × 1 mm^3^) T1-weighted magnetization-prepared rapid gradient echo (MPRAGE) sequence covering the whole brain.

Different imaging orientations were used, with and without the presence of the TMS coil, to evaluate the passive image artifacts. Initially, images were acquired with the bottle phantom in the three orthogonal orientations: axial, coronal, and sagittal for both TMS conditions (with and without TMS coil). Following images of the human brain were acquired in axial and oblique (axial 30° rotation on the x-axis direction, as well as tilted on the y- and z-axis direction to follow the head orientation – Supplementary Figure S3) orientations with the TMS coil, but only the oblique images were acquired without the TMS coil. Finally, images in the oblique orientation were acquired on the bottle phantom to evaluate the active artifacts.

### TMS

The MRI-compatible TMS coil (Air Cooled Coil MRI-B91, MagVenture Inc., Alpharetta, GA) was appended to the MRI-compatible TMS holder (MagVenture Inc., Alpharetta, GA), which was attached to the MRI bed. This holder allows to position the TMS coil inside the Tx/Rx coil, which has a cylindrical shape, through the back of the rf coil. The TMS coil was connected to the stimulator (MagPro X100, MagVenture Inc., Alpharetta, GA) seated outside the MRI scanner room, through a long cable passing through the waive-guide on the filter wall of the scanner room.

#### Imaging With the Phantom (Passive and Active TMS-Induced Image Artifacts)

**FIGURE 1:**
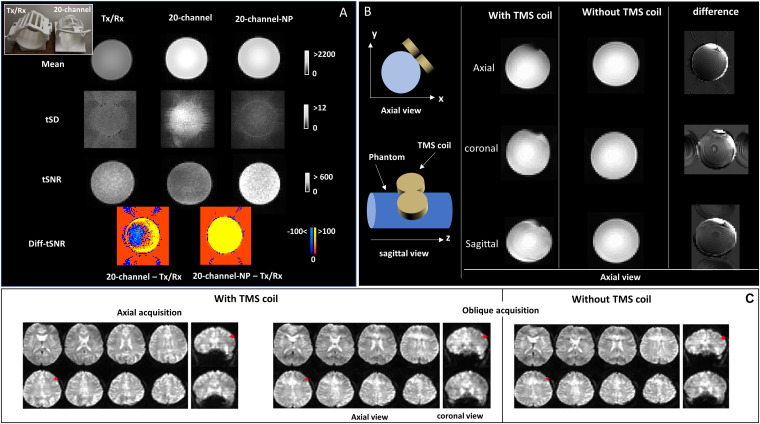
**(A)** A picture of the Tx/Rx and 20-channel coils at the top-left corner. MRI signal intensity (mean), temporal standard deviation (tSD), and temporal signal-to-noise ratio (tSNR) images are shown for echo-planar imaging (EPI) images of the bottle phantom acquired with both rf coils (Tx/Rx and 20-channel); tSNR differences between 20-channel acquisitions (with and without parallel imaging) and Tx/Rx acquisitions (Diff-tSNR) are also shown; NP, no parallel imaging. **(B)** Schematic design of the transcranial magnetic stimulation (TMS) coil positioned over the left side of the phantom, oblique to the xy-plane. Axial view of the mean EPI images is displayed for the axial, sagittal, and coronal data acquisition of the bottle phantom with and without the TMS coil. The difference images (without - with TMS coil) are also displayed. **(C)** Brain EPI images for the axial and oblique data acquisition acquired with the TMS coil, positioned over the left dorsolateral prefrontal cortex (DLPFC, MNI = -50,30,36, highlighted in red), in addition to the oblique acquisition without the TMS coil are shown, on coronal and axial views.

In the MRI suite, the MRI-compatible TMS coil was positioned over the left side of bottle phantom oblique to the xy-plane as displayed on Figure 1B, to mimic the coil position intended to be used during the brain imaging.

#### Imaging With the Participant Head (Passive TMS-Induced Image Artifacts)

In the TMS room, a TMS cap (BrainsWay Ltd.) was placed over the participant head. The left dorsolateral prefrontal cortex (DLPFC) was selected as the stimulation target at the Montreal Neurological Institute (MNI) coordinate: [–50,30,36] using the BrainSight TMS Navigation System (Rogue Resolutions Ltd., United Kingdom) and the C-B60 figure-of-eight TMS coil (MagVenture Inc., Alpharetta, GA). This locus was then marked on the surface of the TMS cap as indicator for TMS coil positioning in the MRI suite. The left DLPFC was chosen because it is a preferential site used for treating depression and addition (Polley et al., 2011; Terraneo et al., 2016), and there is increasing interest in understanding the underlying mechanism of TMS in this area.

In the MRI suite, the MRI-compatible TMS coil was positioned over the left DLPFC (MNI coordinates: [-50, 30, 36]) as marked at the TMS cap. No TMS pulse was applied over the participant head at any time, as this step was conducted for testing the passive artifact only. No fiducial marker was used.

### Stimulation Paradigm (Active TMS-Induced Image Artifacts)

**FIGURE 2:**
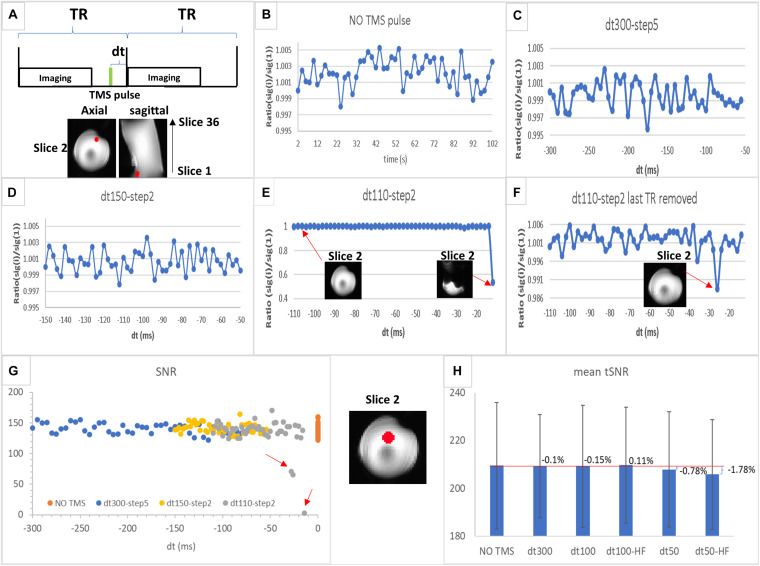
**(A)** Schematic illustration of interleaved transcranial magnetic stimulation (TMS) and functional MRI (fMRI) data acquisition. dt is the time interval between TMS pulse (in green) and imaging acquisition block; TR is the repetition time. **(B–F)** Region of interest (ROI) values of MRI signal (ratio between value for each time point and the value for the first one) in a sphere (6 mm radius, in red) located at the slice 2 (the first slice acquired in an even number of slices acquisition mode) for experiments 1–4 as follows: **(B)** echo-planar imaging (EPI) acquisition without TMS pulse (experiment 1); **(C)** EPI acquisition with TMS applied at a rate of 1 pulse per volume with dt varying between 300 and 50 ms at a step of 5 ms (experiment 2); **(D)** EPI acquisition with TMS applied at a rate of 1 pulse per volume with dt varying between 150 and 50 ms at a step of 2 ms (experiment 3); **(E)** EPI acquisition with TMS applied in a rate of 1 pulse per volume with dt varying between 110 and 10 ms at a step of 2 ms (experiment 4) showing one image as an example of those unaffected by TMS and another from the last time point strongly affected by TMS pulse; **(F)** the same as **(E)** but without the last time point (dt = 10 ms). **(G)** Signal-to-noise ratio (SNR) values in the ROI (sphere of 12 mm radius highlighted in red displayed between **G,H**) for each time point in the time series are plotted for each experiment shown in **(B–E)** [SNR values for NO TMS **(B)** are plotted at dt = 0]. The red arrows highlight those time points with SNR values lower than the normal range determined by the NO TMS run; **(H)** mean tSNR values in the same ROI as **(G)** for NO TMS (experiment 1), dt300 (experiment 5, single-pulse TMS followed by imaging with dt of 300 ms), dt100 (experiment 6, single-pulse TMS followed by imaging with dt of 100 ms), dt50 (experiment 7, single-pulse TMS followed by imaging with dt of 50 ms), dt100-HF [experiment 8, 5 pulses (10 Hz) TMS followed by imaging with dt of 100 ms; HF, high-frequency], and dt50-HF [experiment 9, 5 pulses (10 Hz) TMS followed by imaging with dt of 50 ms]. The bar graph displays the mean tSNR values in the ROI with the percent change as defined in Eq. 3 for each TMS experiment compared with the baseline (NO TMS), the error bar displays the SD of the tSNR values inside the ROI.

Several TMS stimulation paradigms were used to evaluate TMS-induced active image artifacts. Air cooling was used at all times. Active image artifacts were evaluated only with the bottle phantom, since these artifacts are not dependent on the type of sample being imaged. The experiment was designed to find the optimum time interval between TMS pulse and imaging acquisition (dt in Figure 2A). TMS intensity was kept at 50% of maximum stimulator output (MSO). For this purpose, 50 images were acquired on each experiment, and TMS was applied before each image for different values of dt as follows:

1. EPI volumes were acquired without TMS stimulation as baseline.
2. EPI volumes were acquired at a rate of 1 TMS pulse per volume with dt varying between 300 and 50 ms at a step of 5 ms.
3. EPI volumes were acquired at a rate of 1 TMS pulse per volume with dt varying between 150 and 50 ms at a step of 2 ms.
4. EPI volumes were acquired at a rate of 1 TMS pulse per volume with dt varying between 110 and 10 ms at a step of 2 ms.
5. EPI volumes were acquired at a rate of 1 TMS pulse per volume with dt of 300 ms.
6. EPI volumes were acquired at a rate of 1 TMS pulse per volume with dt of 100 ms.
7. EPI volumes were acquired at a rate of 1 TMS pulse per volume with dt of 50 ms.
8. EPI volumes were acquired at a rate of 5–10 Hz TMS pulses (5 pulse, with interpulse interval of 0.1 s or 10 Hz) per volume with dt of 100 ms.
9. EPI volumes were acquired at a rate of 5–10 Hz TMS pulses per volume with dt of 50 ms.

### Data Processing

All data analyses were carried out using the software package of Analysis of Functional NeuroImages (AFNI) (Cox, 1996).

#### rf Coil Comparison

In order to compare image quality between the two head coils, a set of voxel-wise metrics were calculated: the average MRI signal across the time series in each voxel [mean(i), i = voxel index], the temporal standard deviation in each voxel [tSD(i)], and the temporal signal-to-noise ratio in each voxel [tSNR(i)] defined as:

(1) $$\text{tSNR}\,\left(\text{i}\right)=\frac{\text{Mean}\,\left(\text{i}\right)}{\text{tSD}\,\left(\text{i}\right)}$$

Direct comparison of tSNR between images acquired with the Tx/Rx rf coil and those acquired with the 20-channel rf coil (IPAT ON and OFF) was carried out by calculating the voxel-wise tSNR differences. For this purpose, the tSNR maps, calculated from images acquired with the 20-channel coil, were aligned with the tSNR from those obtained with the Tx/Rx coil, and a direct subtraction of the tSNR maps was performed.

#### Passive Artifacts

To evaluate the extent of the artifacts induce by the presence of the TMS coil inside the rf coil, even when no TMS pulse is applied, the temporal mean MRI images (averaged across the TR volumes) were calculated for each condition (with and without the TMS coil) from the images acquired with the bottle phantom, for each slice orientation (axial, coronal, and sagittal) used in data acquisition. Then, the difference between these temporal mean MRI images (image without TMS coil—image with TMS coil) was computed. For the human brain, the MRI images were aligned and normalized to the MNI template. The first images in the time series were displayed for comparison. A region of interest (ROI) (sphere of 6 mm radius) centered at the left DLPFC (MNI coordinates: [-50, 30, 36]) was created to display the closest brain area underneath the TMS coil center.

#### Active Artifacts

The ratio between the mean MRI signal of each image in the time series [sig(i), i = image index] and the mean signal of the first image in the time series [sig(1)] both extracted from a small ROI (sphere of 6 mm radius shown in red in Figure 2A) was plotted over time for experiments 1–4 to evaluate any small signal variation underneath the coil. Then, the SNR was calculated in a larger ROI (sphere of 12 mm radius shown in red between Figures 2G,H) for these experiments, as defined in the equation below to evaluate any possible SNR variation averaged in a larger bright area of the phantom that may not display signal drop-off.

(2) $$\text{SNR}=\frac{\text{mean}}{\text{SD}\,\left(\text{diff}\right)}\;$$

where mean is the mean MRI signal in the ROI for each image in the time series, averaged across the TR volumes; the SD(diff) is the standard deviation in the ROI for each pair of difference image [Image(j) - Image(j + 1), j = 1…N - 1, N = number of images in the time series].

For experiments 1, 5–9, the tSNR value per voxel was calculated, as defined on Eq. 1. Then, the mean tSNR and respective SD values were extracted in an ROI (the same sphere of 12 mm radius referred above for the SNR calculation) for these experiments. The percentage change (% change) contrasting the experiments with TMS pulse against the baseline (without TMS pulse) was also calculated as follows:

(3) $$\%\mathrm{change}=100*\frac{\left[\text{tSNR}\,\left(\text{TMS}\right)-\text{tSNR}\,\left(\mathrm{NO}\,\mathrm{TMS}\right)\right]}{\text{tSNR}\,\left(\mathrm{NO}\,\mathrm{TMS}\right)}\;$$

where tSNR (TMS) is the mean tSNR values for experiments 5–9, and tSNR (NO TMS) refers to the mean tSNR value in the ROI for experiment 1. For experiment 8, only the first 45 volumes in the time series were considered, since TMS stopped by that volume.

## Results

### rf Coils Comparison

The results of average signal intensity (mean), tSD, and tSNR from EPI images acquired with the bottle phantom using the Tx/Rx and 20-channel (with and without parallel imaging) rf coils, with the purpose of comparing imaging quality from the two coils, are shown in Figure 1A. Signal intensity was higher in the phantom images acquired with the 20-channel coil compared with those acquired with the Tx/Rx-coil (Figure 1A). Image specificity, on the other hand, showed to be comparable for both coils, as observed in images acquired with the ACR phantom that contains fine structures (Supplementary Figure S1, top panel). The spatial distribution of the temporal noise, measured as tSD, showed to be non-uniform across the phantom for the images acquired with the 20-channel coil, leading to heterogeneous distribution of tSNR values as well, while images acquired with the Tx/Rx coil showed to have more uniform tSD and tSNR values across the phantom (Figure 1A and Supplementary Figure S1). Finally, tSNR values from images acquired with the 20-channel coil without parallel imaging (20-channel NP) showed to be higher than the tSNR from images acquired with the Tx/Rx coil, but when parallel imaging was used, some areas of the phantom showed lower tSNR values (Figure 1A).

### Passive Artifacts

Figure 1B displays the results for EPI images of the bottle phantom, with and without the TMS coil positioned over it, acquired with the Tx/Rx coil in different image orientations. The difference images (without—with TMS coil) are also displayed. Distortions in EPI images, related to the presence of the TMS coil, were smallest in phantom images acquired with the axial orientation (Figure 1B and Supplementary Figure S2), whereas distortions in EPI (Figure 1C and Supplementary Figure S3) and anatomical (Supplementary Figure S4) axial/oblique images, of the human brain, was unnoticeable, possibly due to the space between the TMS coil and the cortex surface.

### Active Artifacts

Results for different intervals between the TMS pulse and image acquisition (dt) (Figures 2B–F) indicated that substantial MRI signal variations may occur for dt < 30 ms, since a large reduction in signal intensity from the baseline was observed (Figures 2B,E,F). This signal drop-off may either be accompanied by a strong artifact (Figure 2E) or not be observed in the image (Figure 2F). However, further quantitative evaluation showed a substantial decrease in SNR on images acquired with dt < 50 ms (Figure 2G). In addition, a small decrease in tSNR for dt = 50 ms [low-frequency (single-pulse/image) and high-frequency (5–10 Hz pulses/image)] was observed, with nearly no tSNR change for dt ≥ 100 ms (for both TMS frequencies), compared with the baseline, although all observed tSNR differences were within the dispersion of the tSNR values inside the ROI (Figure 2H).

These results indicate that TMS pulses may still affect the SNR and tSNR, when dt is short (<100 ms), in phantom areas where signal drop-off is not evident; therefore, compromising fMRI results. Finally, these findings suggest that an interval between TMS pulse and imaging acquisition larger than 100 ms will guarantee absence of TMS artifacts.

## Discussion

### Comparison of rf Coils

A direct comparison between images acquired with two different rf coils was carried out to evaluate the pros and cons of using the birdcage coil. The 20-channel rf coil provided images not only with higher MRI signal but also with higher and spatially heterogeneous temporal noise, leading to a non-uniform distribution of tSNR in the images. The single-channel birdcage coil, on the other hand, provided lower signal intensity but more homogeneous noise across the image. Besides, image specificity, in terms of resolving small structures, was comparable between the two coils. Overall, the birdcage coil showed to be suitable for multimodal TMS and MRI studies, despite the lack of advanced features such as parallel imaging and multiband/multislices acquisition with this coil.

The combination of two seven-channel surface coils (Navarro de Lara et al., 2015) has been proposed for simultaneous TMS and fMRI; however, this approach showed to be highly non-uniform and overall low SNR (de Weijer et al., 2014). Therefore, while the setup with surface coils may have advantages to detect TMS-induced brain activity near the coils (where tSNR is high), the setup with the birdcage coil is appropriate for the measurement of whole brain activity on simultaneous TMS-fMRI studies.

Our experience in implementing the concurrent TMS-fMRI setup indicates that most of whole-brain multichannel coils do not have the needed space to fit the TMS coil and holder inside, along with the participant head. Whereas the birdcage coil provides enough space for all, allowing the position of the TMS coil in several brain regions. Importantly, the birdcage coil offers suitable sensitivity and specificity for fMRI studies.

### Passive Artifacts

Phantom imaging results showed that imaging orientation is crucial to reduce the susceptibility artifacts induced by the TMS coil, consistent with previous findings (Baudewig et al., 2000; Bestmann et al., 2003a). Imaging distortion and signal loss in the phantom were the main artifacts observed, as reported by Baudewig et al. (2000). The absence of artifacts observed in the human brain, in contrast to those observed with the phantom, suggests that the shape of the object to be imaged and distance from the TMS coil are important factors. While the cylindrical phantom has a larger surface of contact with the TMS coil, the brain, on the other hand, is more spherical and has much reduced surface of contact with the TMS coil. Therefore, the signal loss under the coil area is minimized. Moreover, the distance from the coil to the phantom, which was closer than the distance from the coil to the brain (cortex surface), may also contribute to the larger artifacts observed on the images from the phantom. Passive image artifacts in the human brain were reported by Bungert et al. (2012), and passive shimming was suggested as a solution. However, these artifacts were not observed by Baudewig et al. (2000). Here, we did not observe signal loss underneath the coil, as well, which may be related to the TMS coil position and orientation, the shape of the object to be imaged, and the distance of it to the TMS coil. Our findings are also in agreement with Navarro de Lara et al. (2017), which did not observe any passive artifact (distortion or signal dropout) under the TMS coil, when combining it with the seven-channel surface coil.

### Active Artifacts

It is essential to determine the optimal time interval between the TMS pulse and the beginning of imaging acquisition for implementation of the interleaved TMS and fMRI. With a pulse width of 280 ms, TMS can still interfere with data acquisition. TMS pulses may induce eddy currents in the conducting structures of the MRI system, producing transient magnetic fields and disrupting the transverse magnetization of the sample. When affecting RF excitation, TMS pulses can alter the steady-state longitudinal magnetization (Bestmann et al., 2003a). As a result, image artifacts, signal drop-off, or tSNR variation may occur. Besides, tSNR and SNR may be affected by the TMS pulse even when either image artifacts or signal drop-off is not obvious. For this reason, the approach of removing the disrupted image and replacing it with interpolated data (Heinen et al., 2011; Hawco et al., 2017) may not be optimal, since the tSNR/SNR on non-obvious disrupted images may be affected as well if the TMS pulse was close, compromising fMRI signal. Therefore, a proper time interval between TMS and imaging is needed to avoid the interference of the TMS to MRI.

Here, we found that an interval >50 ms between the TMS pulse and imaging acquisition may be sufficient to avoid active artifacts, but an interval of 100 ms was a safer threshold, consistent with the previous finding (Bestmann et al., 2003a; Navarro de Lara et al., 2017).

In conclusion, in this work, we provided an update on factors related to imaging quality that are important for simultaneous TMS and fMRI. We demonstrated that good quality whole-brain imaging can be acquired using a single-channel rf coil when interleaving with TMS but with the cost of losing more advanced features, such as parallel imaging and multiband/multislices acquisition. Imaging orientation should be optimized in order to substantially reduce potential susceptibility artifacts, since the optimal orientation depends upon the chosen TMS target. Proper time interval between the TMS pulse and MRI acquisition should also be re-evaluated when the technique is implemented. Finally, this work shows the feasibility and effectiveness of this multimodal technique and provides a guideline for implementing this promising neuroimaging tool.

## Data Availability Statement

The datasets presented in this article are not readily available because the raw data supporting the conclusions of this article will be made available by the authors upon request. Any human data will become available following local institutional regulations, where data was generated. Requests to access the datasets should be directed to the corresponding author.

## Ethics Statement

The studies involving human participants were reviewed and approved by the National Institute on Drug Abuse institutional review board. The patients/participants provided their written informed consent to participate in this study.

## Author Contributions

EC: conceptualization, methodology, investigation, formal analysis, visualization, and writing (original draft preparation). TZ: software, investigation, and writing (reviewing and editing). YY: supervision, resources, funding acquisition, and writing (reviewing and editing). All authors contributed to the article and approved the submitted version.

## Conflict of Interest

YY is serving as an Associate Editor of the Frontier in Neuroscience. The remaining authors declare that the research was conducted in the absence of any commercial or financial relationships that could be construed as a potential conflict of interest.
