# Supplementary material for: Simultaneous Transcranial Magnetic Stimulation and Functional Magnetic Resonance Imaging: Aspects of Technical Implementation
Source: Front Neurosci. 2020 Sep 29;14:554714. doi: 10.3389/fnins.2020.554714 (PMC7550427; doi:10.3389/fnins.2020.554714)
Supplement: Supplementary file 1 [file Table_1.DOCX]

**Simultaneous transcranial magnetic stimulation and functional magnetic resonance imaging: aspects of technical implementation**

Elisabeth C. Caparelli^1^, Tianye Zhai^1^ and Yihong Yang^1^

1. Neuroimaging Research Branch, National Institute on Drug Abuse, National Institutes of Health, Baltimore, Maryland, USA

**Supplemental Material**

The mean MRI signal in the images acquired with the 20-channel coil, showed to be higher than the mean MRI signal in the images acquired with the Tx/Rx-coil (Figure 1A and S1 (Supplemental material) top). Basically, images acquired with a multi-channel rf-coil have high signal intensity on the peripheral areas of the imaged object, since those areas are closer to the coil receivers. During data reconstruction the high signal intensity is spread out through the image after signal normalization, raising the MRI signal of the entire image, mainly due to the pre-scan normalize option. On the other hand, the image acquired with the Tx/Rx-coil is not normalized, being the homogeneous distribution of the MRI signal through the phantom a sole result of the acquisition method.

Image specificity seems to be comparable on both coils, since the different geometrical figures presented within the ACR-phantom were closely reproduced on the images acquired with both coils (Figure S1 top). TSNR values showed to have an irregular distribution for images acquired with the 20-channel coil (IPAT ON), but be more homogeneous distributed for those acquired with the Tx/Rx coil (Figures 1A and S1 bottom).


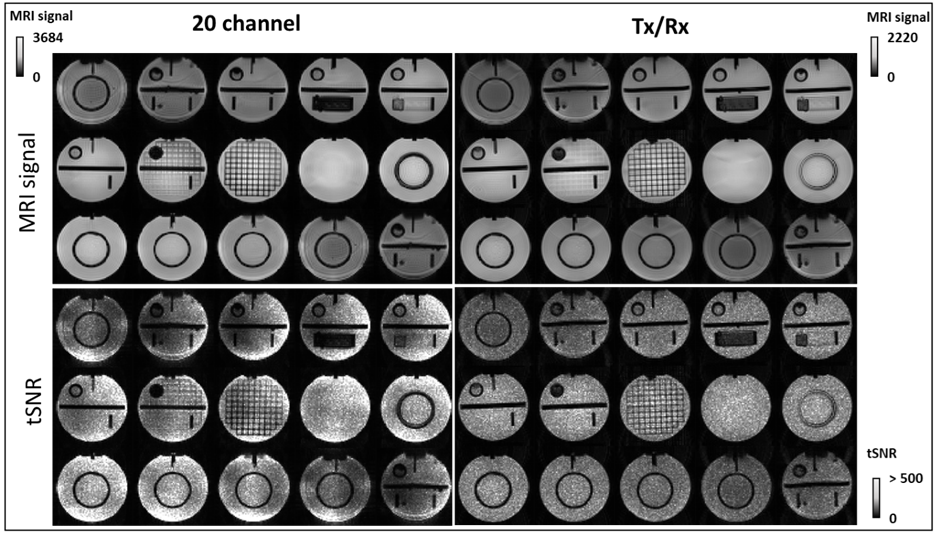


Figure S1: MRI signal for the first image in the respective time series and tSNR values are shown for the ACR-phantom acquired with the 20-channel (IPAT ON) and the Tx/Rx rf-coils.


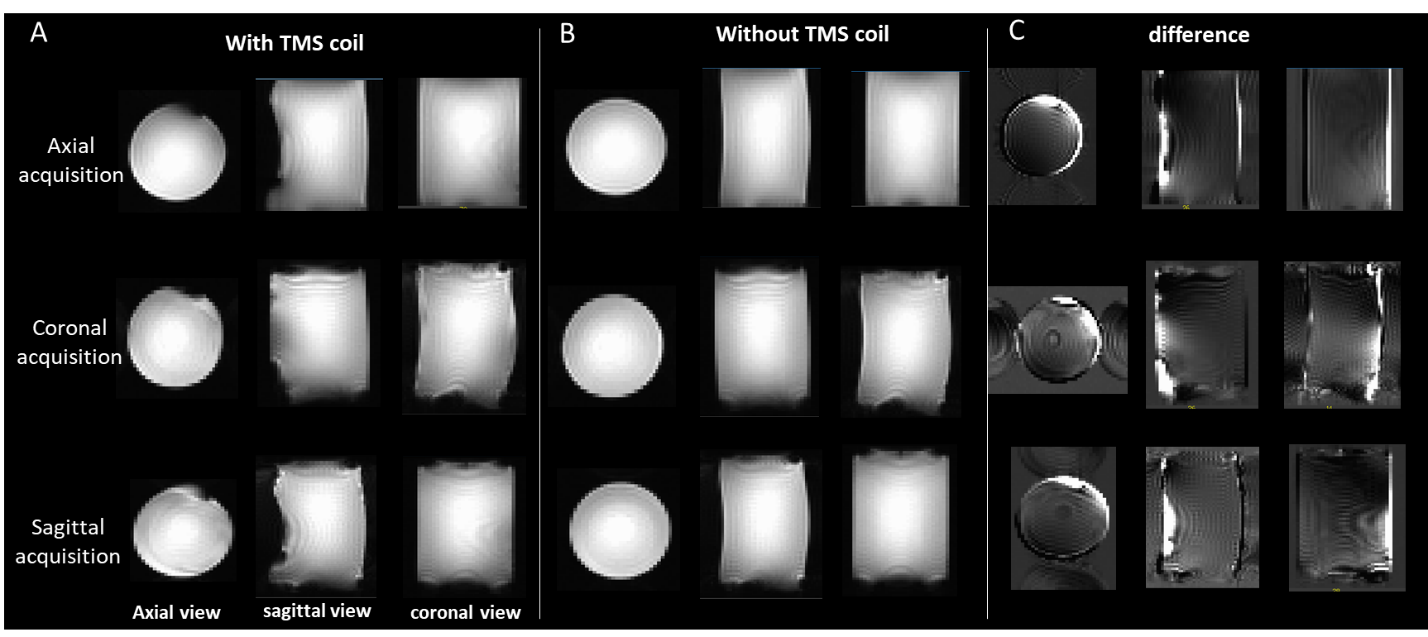


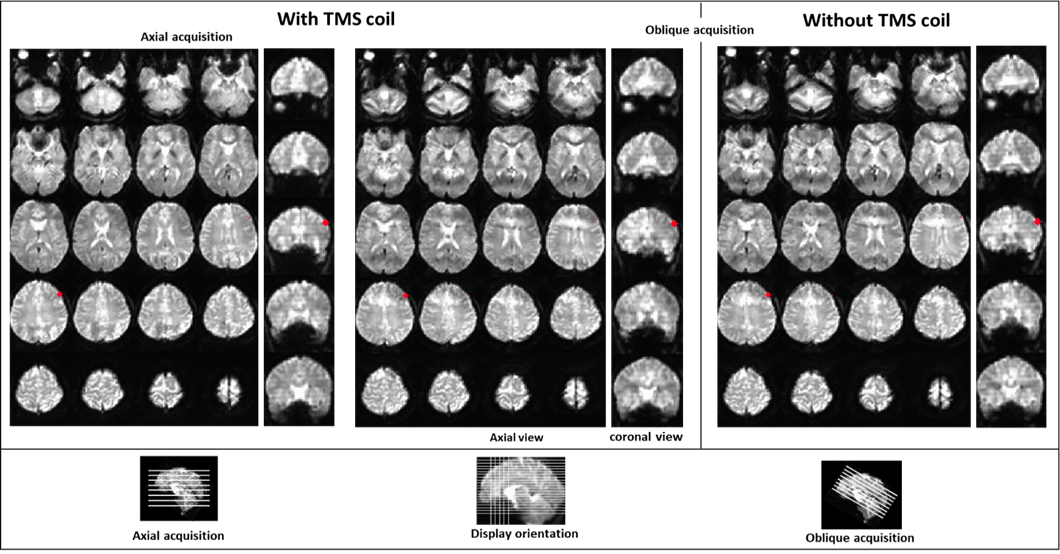
Figure S2: Axial, sagittal and coronal views are displayed for the axial, sagittal and coronal acquisition of bottle-phantom with (A) and without (B) the TMS coil when positioned on the left side of the phantom (Figure 1B). The difference (B-A) is also displayed (C).

Figure S3: EPI images of the brain for axial and oblique acquisitions with the TMS coil and for oblique acquisition only without the TMS coil. The red circle shows the TMS coil position, over the left DLPFC (MNI = -50,30,36). Sagittal view shows the slice location for the axial and coronal views.


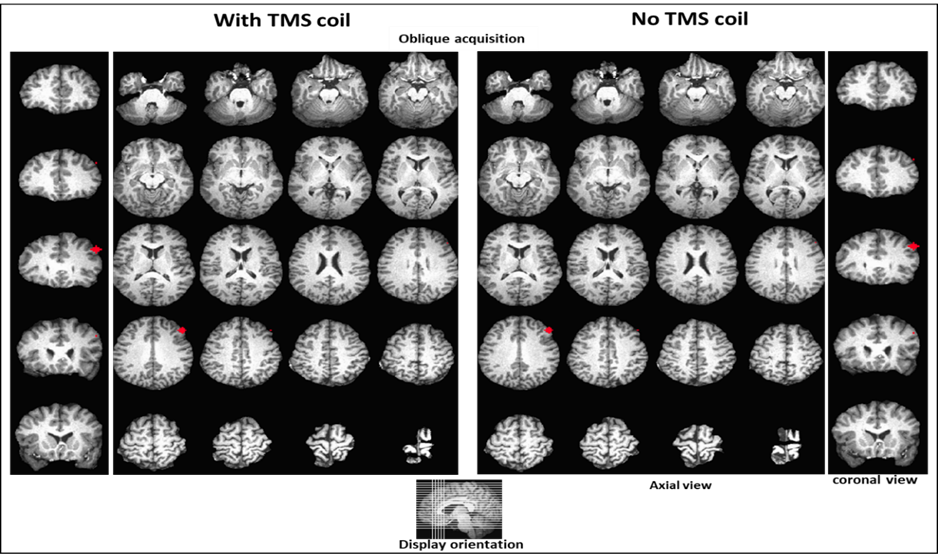


Figure S4: Anatomical images of the brain for the oblique acquisition (as displayed on figure S3) are shown, on coronal and axial views with and without TMS coil. Sagittal view shows the slice location for the axial and coronal views. TMS coil position over the left DLPFC (MNI = -50,30,36), is highlighted in red on coronal and axial views.
